# First natural crossover recombination between two distinct species of the family *Closteroviridae* leads to the emergence of a new disease

**DOI:** 10.1371/journal.pone.0198228

**Published:** 2018-09-13

**Authors:** Leticia Ruiz, Almudena Simón, Carmen García, Leonardo Velasco, Dirk Janssen

**Affiliations:** 1 IFAPA Centro La Mojonera, IFAPA, La Mojonera, Almería, Spain; 2 IFAPA Centro Churriana, IFAPA, Churriana, Málaga, Spain; USDA Agricultural Research Service, UNITED STATES

## Abstract

*Lettuce chlorosis virus*-SP (LCV-SP) (family *Closteroviridae*, genus *Crinivirus*), is a new strain of LCV which is able to infect green bean plants but not lettuce. In the present study, high-throughput and Sanger sequencing of RNA was used to obtain the LCV-SP full-length sequence. The LCV-SP genome comprises 8825 nt and 8672 nt long RNA1 and RNA2 respectively. RNA1 of LCV-SP contains four ORFs, the proteins encoded by the ORF1a and ORF1b are closely related to LCV RNA1 from California (FJ380118) whereas the 3´ end encodes proteins which share high amino acid sequence identity with RNA1 of *Bean yellow disorder virus* (BnYDV; EU191904). The genomic sequence of RNA2 consists of 8 ORFs, instead of 10 ORFs contained in LCV-California isolate. The distribution of vsiRNA (virus-derived small interfering RNA) along the LCV-SP genome suggested the presence of subgenomic RNAs corresponding with HSP70, P6.4 and P60. Results of the analysis using RDP4 and Simplot programs are the proof of the evidence that LCV-SP is the first recombinant of the family *Closteroviridae* by crossover recombination of intact ORFs, being the LCV RNA1 (FJ380118) and BnYDV RNA1 (EU191904) the origin of the new LCV strain. Genetic diversity values of virus isolates in the recombinant region obtained after sampling LCV-SP infected green bean between 2011 and 2017 might suggest that the recombinant virus event occurred in the area before this period. The presence of LCV-SP shows the role of recombination as a driving force of evolution within the genus *Crinivirus*, a globally distributed, emergent genus.

## Introduction

*Lettuce chlorosis virus* (LCV) belongs to the genus *Crinivirus*, family *Closteroviridae*. Viruses included in this family are the largest among the known plant viruses and present ssRNA, positive-sense genome [1]. Family *Closteroviridae* has been classified into three genera based on vector transmission and phylogenetic relationships: *Closterovirus*, *Ampelovirus*, and *Crinivirus*. Recently, a new genus named *Velarivirus* has been proposed [2]. All members of genus *Crinivirus* include segmented genomes, are whitefly-transmitted and limited to the phloem [3].

Many members of genus *Crinivirus* are considered emerging epidemics worldwide [4]. LCV was described in the 1990´s in California, transmitted by the whitefly *Bemisia tabaci* and affecting lettuce and sugar beet crops [5]. The complete sequence and the genomic organization of LCV were described in 2009 [6] and comprises a bipartite genome formed by RNA1 and RNA2. LCV RNA1 contains the replication module (ORFs 1a and 1b), which encodes conserved domains of a papain-like leader proteinase (P-PRo), a methyltransferase (MTR), helicase (HEL) and RNA-dependent RNA polymerase (RdRp), ORF2 encodes a putative 8-kDa protein (P8), and ORF3 encodes a 22.9-kDa protein (P23) recently described as viral suppressor of RNA silencing [7]. LCV RNA2 contains the hallmark *Closterovirus* gene array coding for a heat shock protein homolog (Hsp70h, ORF3), a 50–60 kDa protein (ORF5), the major (CP, ORF7) and the minor coat protein (CPm, ORF 8) [1,8]. Furthermore, P5.6, P6, P6.4, P9, P27, and P4.8 are encoded by ORF1, ORF2, ORF4, ORF6, ORF9 and ORF10, respectively; the functions of these putative proteins remain unknown [6].

LCV shows high sequence similarity with *Cucurbit chlorotic yellow virus* (CCYV) and with *Bean yellow disorder virus* (BnYDV), [6,9]; the latter one recognized as the first *Crinivirus* that infects Leguminosae family crops [10]. LCV was considered as an endemic infection which had not spread to areas outside Southwestern USA [4]. In 2014 however, symptoms similar to BnYDV in green bean crops were attributed to a new strain of LCV, LCV-SP, which was unable to infect lettuce plants and capable of infecting green bean crops [11]. Recently, the two Chinese isolates of LCV (LCV-Nj and LCV-CN) infecting Nicotiana species and ornamental plants were fully sequenced. Both RNA1 and RNA2 genome have 85 and 88% nucleotide sequence identity with those of LCV from California [12,13].

Viral RNA-RNA recombination and/or reassortment of genomic segments (pseudo-recombination), between virus strains or virus species, increase the genetic variability and is a powerful process in virus evolution, responsible for the emergence of new viral strains or species [14]. RNA recombination can also be a process to purged deleterious mutations from virus populations accumulated due to the high mutation rates of RNA viruses, which would avoid fitness loss in small viral populations [15]. The low frequency of its detection is probably the result of selection pressure that removes the majority of recombinants [16]. The first evidence of genetic recombination was described in *Bromo mosaic virus* [17] and it seems to be specially frequent among species belonging to the family *Potyviridae* where crossovers have been described at ORFs and 3´UTR sequences [18–21,16].

Within the family *Closteroviridae*, homologous recombination has been described at CP, P20 and in defective RNAs and sgRNAs in the *Closterovirus* member *Citrus tristeza virus* (CTV) [[22–24] and at CP of the *Ampeloviruses Grapevine leafroll-associated virus* 3 (GLRaV-3) and GLRaV-11 [24,25]. Although *Closterovirus* members share a core of conserved genes, they present high variability in the set of ORFs located in the 3´end of RNA or in the 3´end of RNA 1 if the genome is bipartite, as is the case of the genus *Crinivirus* [1,26]. Some genes in the 3`region of genus *Crinivirus* have been described to encode RNA silencing suppressor (RSS) proteins. The presence of mechanisms capable of increasing genetic variability such as genome recombination may have important evolutionary implications because some of these proteins have been characterized as RSS proteins [7, 27–30].

Plant viruses are strong inducers and targets of RNA silencing, and generally high levels of virus-derived small interfering RNAs (vsiRNAs) are accumulated during virus infections. Characterization of vsiRNAs by deep sequencing has primarily been carried out in experimental host plants and less so in horticultural species [31].

In this study we describe a recombinant *Crinivirus*, LCV-SP, originating from a crossover of intact ORFs between LCV-RNA1 and BnYDV-RNA1 as parentals, and analyse a population of isolates collected in the area. The implications in emergence of *Crinivirus* epidemiology are discussed.

## Material and methods

### Virus isolate

The owners of the orchards granted permission to collect the samples

Between 2011 and 2017, samples of green bean leaves showing symptoms that could be attributed to LCV-SP (interveinal mottling and yellowing in lower and middle leaves [11]) were collected from different geographical locations from Granada, Almeria and Málaga provinces in Southeastern Spain. The plants were analysed by RT-PCR to ensure the presence of LCV-SP [11] and stored at -80°C for subsequent molecular analysis. One of these isolates collected in 2013 and annotated as "Almeria" was used for complete genome determination.

### Whole-genome sequencing of LCV-SP and genome analysis

Total RNA from LCV-SP isolate "Almeria" was extracted with Trizol Reagent (Invitrogen) according to the manufacturer’s instructions. Partial length viral sequence of RNA1 and RNA2 of LCV genome was determined by RT-PCR using a primer walking strategy with a set of degenerate primers based on LCV (FJ380118, FJ380119) and BnYDV (NC010560, NC010561) sequences. Partial sequences of LCV-SP RNA1 and RNA2 previously described [11] were also used to design specific primers and flank the gaps. The sequences were assembled using the software GENEIOUS (v. 7.1.9, Biomatters, New Zealand).

### Small RNA purification, dsRNA extraction and deep sequencing

Small RNA purification from the Almeria LCV-SP isolate infected tissue was obtained using the miRCURY RNA isolation kit (Exiqon, Denmark), according to the manufacturer’s instructions. Small RNA library was prepared using the Illumina TruSeq Small RNA Kit (Illumina, San Diego, California, USA). The library was sequenced on the Illumina deep sequencing platform using the services provided by the Centre for Genomic Regulation (CRG, Barcelona, Spain). Sequencing adapters and reads shorter than 18 nucleotides (nt) were removed using GENEIOUS and reads ranging from 18 to 24 nt were selected. These reads were assembled into contigs larger than 33 nt using the *de novo* assembly function of VELVET v. 1.2.08 implemented in the SCBI Picasso server (http://www.scbi.uma.es) with a k-mer = 18. Partial RNA1 and RNA2 LCV-SP sequence obtained for Sanger sequencing was used as a reference for contigs sequences. RNA1 and RNA2 consensus draft sequences obtained were ensured through RT-PCR amplification using specific primer pair based on the assembled genomes (not shown). The 5´/3´ Smart^TM^ RACE cDNA Amplification Kit (Clontech, California, USA) was used to complete the 5´ and 3´ends. PCR fragments were cloned in pGEM-T vector and at least two cDNA clones were sequenced in both directions. To obtain the profile distribution of LCV-SP virus-derived small interfering RNAs (vsiRNAs), the 21 and 22 nt reads of the small RNA library were aligned to the assembled LCV-SP genome using MISIS-2 [32].

### Double stranded RNA purification

Deep sequence from LCV-SP dsRNA was used to confirm the LCV-SP RNA1 and RNA2 genomic sequences. For that, double-stranded RNA was purified from another LCV-SP isolate collected in 2014 [33] followed by S1 nuclease (Promega, Madison, Wisconsin, USA) and DNAase I (Invitrogen, Carlsbad, California, USA) treatments to discard the contamination with genomic DNA and ssRNAs. A library was constructed and sequenced using the Illumina deep sequencing platform with the services provided by CRG (Barcelona, Spain). Contigs were assembled after trimming the low-quality reads by using VELVET (k-mer = 31). Complete sequence of LCV-SP genome obtained from dsRNA was used as reference for contigs aligning in GENEIOUS v. 7.1.9.

The contigs obtained from both massive deep sequencing were conducted to BLAST analysis against the GenBank database in NCBI (http://www.ncbi.nlm.nih.gov) using GENEIOUS. The e-value cut-off was set to 10–5 so high confidence matches could be reported. The numbers of reads aligning to LCV or BnYDV genomes were annotated to confirm the presence of sequences belonging to both virus species.

### LCV-SP virion purification

LCV-SP isolate "Almeria" virions were purified after adapting the methodology described for CTV by [34]. For that 7g of virus-infected leaves were ground in liquid nitrogen until powder to which 100 mL 0.05 M Tris-HCl buffer, pH 7.4 was added. Next, the suspension was filtered through miracloth for removing cellular debris and centrifuged for 10 min at 3,000 rcf. Then 4g of polyethylene glycol (PEG 6000) and 4 mL of NaCl (20%) were added to the supernatant followed by slowly stirring overnight at 4°C. The pellet was collected by centrifugation at 17,500 rcf for 15 min and resuspended in 2 mL of 0.04 M sodium phosphate buffer, pH 8. After 10 min centrifugation at 7,500 rcf, the supernatant containing the virions was further purified by centrifugation at 44,500 rcf for 6 h. Finally, the pellet was resuspended in 400 μL of the same phosphate buffer and stored at -80°C until further use. All centrifugation steps were conducted at 4°C using a Universal 32R centrifuge (Hettich, Tuttlingen, Germany). RT-PCR identification of LCV-SP was carried out with 5 μL of the virion preparation using the sets of primers LCVSP-1F/LCVSP-2R and LCVSP-3F/LCVSP-4R previously described to detect the virus [11] and the set of primers LCVSP-54F 5´TCACAGCCGAGATCAACAGAG 3´ (nt 7170–7190) and LC-Bn51R 5´ACAACAGATCAAAATCCACAATG 3´ (nt 7906–7928) which were designed to amply part of the 3’ end of the LCV-SP RNA1 genome corresponding to the partial region of ORF1b and P26 proteins

### Phylogenetic analysis

For phylogenetic analysis, the complete genome sequences from the criniviruses, LCV (FJ380118, FJ380119), *Lettuce infectious yellows virus* (LIYV, NC003617, NC003618), *Cucurbit yellow disorder virus* (CYSDV, NC004809, NC004810), *Cucurbit chlorotic yellow virus* (CCYV, JQ904628, JQ904629), *Bean yellow disorder virus* (BnYDV, EU191904, EU191905), *Beet pseudo yellows virus* (BPYV, AY330918, AY330919), *Tomato chlorosis virus* (ToCV, KP137100, KP137101) and *Potato yellow vein virus* (PYVV, NC006062, NC006063), were retrieved from the GenBank database and compared with the complete sequence of LCV-SP; the genome sequence from the *Closterovirus Citrus tristeza virus* (CTV, NC001661) was used as outgroup. The pairwise percentage of nucleotide sequence identity was performed using the Clustal W algorithm present in MEGA v 7.01 program [35]. Appropriate nucleotide substitution model was determined using jModelTest 2.1 [36] and the best model proposed by the Akaike information criterion (AIC) applied (GTR+I+G for RNA1 and HSP70h). Bayesian consensus phylogenetic trees were inferred using Bayesian inference and Markov chain Monte Carlo (MCMC) simulation implemented in MrBayes plugin. MCMC analyses were run with a chain length of 10^7^ generations, sampling every 1000 trees and with a burn-in of 25% and chain heated to 0.2.

### Recombination analysis

Recombination Detection Program 4 (RDP4) was used to detect potential recombination events in LCV-SP, likely parental sequences, and localization of recombination breakpoints [37]. The program was executed with default parameter settings which include the RDP, GENECONV, Chimaera, MaxChi, BOOTSCAN, and SISCAN methods. For recombination analysis, sequences of RNA1 and RNA2 of LCV, BnYDV, LCV-SP and CCYV were aligned in MEGA v.7.0.1 software [35] and exported to the RDP4 program for recombination analysis. The program was performed using the default settings and a Bonferroni corrected *p*-value cut-off (α = 0.05). Only recombination events detected by four or more methods were considered as significant. The results obtained by RDP4 were confirmed using a boot scanning method in the SimPlot program v.3.5.1 [38].

#### Genetic diversity in the recombinant genomic region

The crossover recombination event was confirmed in several isolates by RT-PCR. Eleven isolates were obtained in the surveys from 2011 (3), 2012 (2), 2013 (1), 2014 (2), 2015 (1) and 2017 (2). They were analysed by RT-PCR after RNA extraction using the primers LCVSP-44F 5´GCATTCAAGAAATTGTGGGATG 3' (nt 7517–7538) and LCVSP-50R 5´ATATTAATGTAATTCTACGGTC 3´ (nt 8738–8759) which amplified a region of LCV-SP encompassing 1242 nt corresponding to the entire region of the P26 and P6 proteins. Reverse transcription of the isolates was carried out with a reverse primer using Superscript II (Invitrogen) at 42°C for 60 min. Subsequently, DNA fragments were amplified by PCR with Expand High Fidelity PCR System (Roche, Basel, Switzerland) with the following conditions: 95°C for 3 min, 35 cycles of denaturation for 20s at 95°C, annealing for 30s at 55°C and extension for 40s at 72°C followed by one final extension cycle for 5 min at 72°C. PCR products were cloned into pGEM-T vector (Promega Corporation, Madison, USA). The clones were bidirectionally sequenced. Nucleotide alignments were performed using Clustal W in MEGA v. 7.01 [35] software with default settings. The aligned sequences were used to calculate the genetic distances for synonymous (dS) and non-synonymous substitution (dNS) as described by [39,40] (PBL method).

### Molecular differentiation between yellowing induced by BnYDV or LCV recombinant virus (LCV-SP)

In order to determine analytically the presence of the recombinant *Crinivirus* LCV-SP, a RT-PCR-restriction fragment length polymorphism (RFLP) was performed. The set of primers LC-Bn56F 5´GATTTTGGATTTGAAGC 3´ and LC-Bn51R (described above) amplified 739 and 769 bp fragments of LCV-SP RNA1 and BnYDV RNA1 genome, corresponding to nt 7190–7928 and 7294–8062, respectively by RT-PCR. Next, the PCR products were digested with *Kpn*I (Promega) following the manufacturer's instructions. Band patterns were analysed on a 2% agarose gel.

## Results

### Genome sequencing of LCV-SP

**Fig 1:**
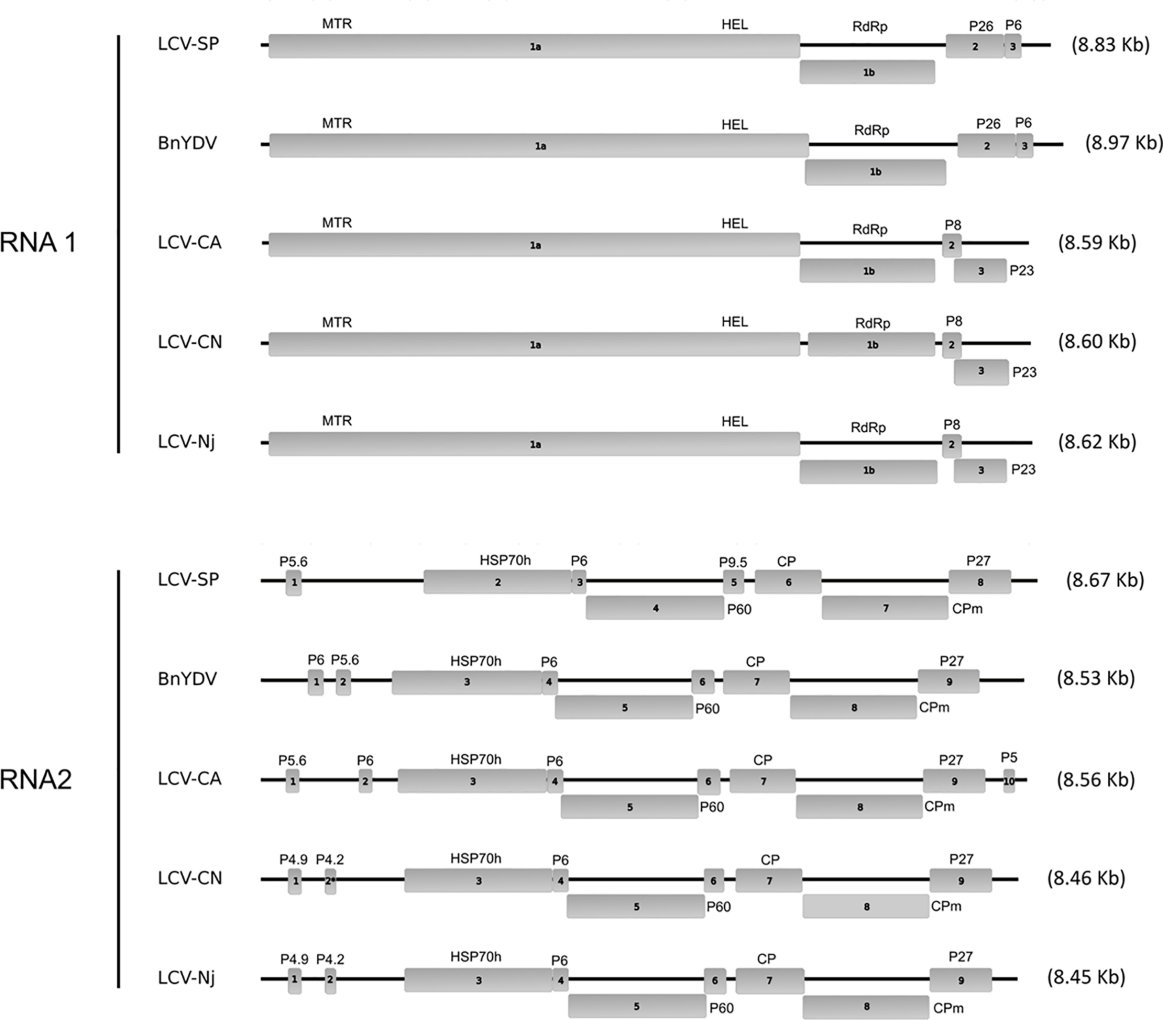
Diagram of genome organization of LCV-SP genome, LCV from California, BnYDV and the Chinese LCV isolates LCV-Nj and LCV-CN. Boxes represent ORFs; putative protein products are also indicated. The asterisk in LCV-CN RNA2 show a P4 protein detected using the software GENEIOUS (v. 7.1.9, Biomatters, New Zealand) which is not present in the original diagram [13].

Walking strategy and Sanger sequencing allowed us to obtain 7109 bp and 7821 bp corresponding to 80% and 90% of complete genome of RNA1 and RNA2 of LCV-SP. The LCV-SP genome was completed by deep sequencing from small RNAs and RACE strategy. The genome was confirmed through RT-PCR amplification with specific primers (S1 Table) and consisting of 8825 nt and 8672 nt equivalent with RNA1 and RNA2 of GenBank accession numbers MG489894 and MG489895, respectively. The genetic organization of LCV-SP genome, LCV from California (FJ380118, FJ380119), BnYDV (EU191904, EU191905) and the Chinese LCV isolates LCV-Nj (KX685958, KX685959) and LCV-CN (KY430285, KY430286) is shown in Fig 1. RNA1 of LCV-SP contains four ORFs: ORF1a (nt 73–6045) and ORF1b (nt 6044–7561) contains the replication module and the P-PRO, MTR and RdRp motifs. ORF2 (nt 7679–8338) encodes a predicted protein of 26-kDa (P26) and it is followed by another putative protein of 6-kDa (P6) (ORF3, nt 8339–8512). The proteins encoded by ORF1a and ORF1b share an amino acid sequence identity of 93.6% and 99.6%, respectively, with LCV RNA1 from California (FJ380118) and a 91% and 99% of amino acid sequence identity with LCV-Nj and LCV-CN (KX685958, KY430285), respectively. Whereas the 5´ terminal sequence from LCV-SP RNA1 is closely related to the RNA1 genome of the Californian (FJ380118) and Chinese isolates (KX685958 and KY430285); the 3´ end encodes proteins which are unique to BnYDV (EU191904) (Table 1). The P26 (ORF2) and P6 (ORF3) share a 99.5% and 100% amino acid sequence identity with those present in BnYDV. Both proteins currently have an unknown function; P26 has no homologues in the GenBank database and P6 display only 30% of amino acid sequence identity with a P6a protein of a new unclassified *Crinivirus* named *Tetterwort vein cholorosis virus* (ALE18214).

**Table 1:** Percentages of amino acid sequence identity of LCV-SP RNA1 (MG489894) with LCV RNA1 (FJ380118) and BnYDV-RNA1 (EU191904).

	ORFs	LCV-SP
**LCV-RNA1**	ORF-1a	93.6
	ORF-1b	99.6
**BnYDV-RNA1**	P26	99.5
	P6	100

The genomic sequence of RNA2 consists of 8 ORFs, instead of the 10 ORFs contained in LCV-California isolate, 9 ORFs as described in LCV-Nj and 8 in LCV-CN (KX685959 and KY430286) [6]. The genomic sequence comprises the hallmark gene array of the family *Closteroviridae* coding for a HSP70h (ORF5, nt 1813–3483), and three diverged copies of the capsid protein: the ortholog of the coat protein homolog (ORF4, nt 3642–5195), the CP (ORF6, nt 5537–6289) and the CPm (ORF7, nt 6289–7713) which exhibit more than 95% amino acid sequence identity with RNA2 of LCV FJ380119. Percentages higher than 93% are shown in the case of both Chinese LCV isolates (KX685959 and KY430286). RNA2 of LCV-SP encodes also three putative small proteins: P5.6 (ORF1, nt 270–422), P6.4 (ORF3, nt 3484–3648) and P9 (ORF5, nt 5177–5416), which show 92%, 100% and 96% nucleic acid identity with those in LCV FJ380119 and 88%, 100% and 96% nucleic acid identity with LCV isolates KX685958 and KY430286. Two putative small proteins in the RNA2 of LCV-Californian isolate (FJ380119), P6 in the 5´ end, and P4.8 in the 3´end, which have unknown function are absent in LCV-SP and in the Chinese LCV isolates. The last ORF of RNA2 (ORF9) encodes a putative P27, which shows a high amino acid identity with its counterpart proteins in the LCV genome sequences in GenBank: 98% with the Californian isolate FJ380119 and 96% with the Chinese isolates KX685958 and KY430286.

#### 5´and 3´ untranslated region

The putative 5’UTR of LCV-SP RNA1 and RNA2 are 72 and 269 nt in length, and share 99% and 96% of nucleotide identity with LCV FJ380118 and FJ380119, respectively. The first 5 nucleotides, GAAAT, are identical in both RNAs. This feature has been described in the Californian LCV isolate and in other members of genus *Crinivirus* [6]. Table 2 shows the percentage of nucleotide identity among 3´UTR regions corresponding with LCV-SP (RNA1 and 2), BnYDV (RNA1 and 2) and the Californian isolate of LCV (RNA 1 and 2). The 3´UTR of RNAs 1 and 2 are 313 and 256 nt in length, respectively, and shared high nucleotide sequence identity (72%). Also, 3´UTR of RNA1 displays almost identical nucleotide sequence (99%) as its equivalent in BnYDV. This sequence is next to the putative proteins P26 and P6 corresponding to the 3´end of RNA1 of BnYDV and LCV-SP. Additionally, 3´UTR of RNA2 of LCV-SP show the highest homology (89%) with the 3´UTR of RNA1 of the Çalifornian isolate of LCV FJ380118.

**Table 2:** Percentage of nucleotide identity between 3´UTRs of LCV-SP, LCV and BnYDV.

	LCV-SP RNA1	BnYDV RNA1	LCV RNA1	LCV-SP RNA2	LCV RNA2
**BnYDV RNA1**	99.7				
**LCV RNA1**	70.1				
**LCV-SP RNA2**	71.4	71.4	89.5		
**LCV RNA2**	72.2	71.4	79.8	79.6	
**BnYDV RNA2**	56.2	57.0	57.8	61.3	52.8

LCV RNA1: FJ380118; LCV RNA2: FJ380119; BnYDV RNA1: EU191904; BnYDV RNA2: EU191905; LCV-SP-RNA1: MG489894; LCV-SP RNA2: MG489895

RT-PCR products, produced from purified LCV-SP virions using the three sets of primers LCVSP-1F/LCVSP-2R, LCVSP-3F/LCVSP-4R and LCVSP-54F/LC-Bn51R yielded fragments of the expected sizes of 434, 463 and 759 bp, corresponding to the partial region of RNA2-HSP70, RNA1-ORF1a and RNA1-ORF1b and P26, respectively (S1 Fig)

### Libraries of small RNAs and dsRNAs

**Fig 2:**
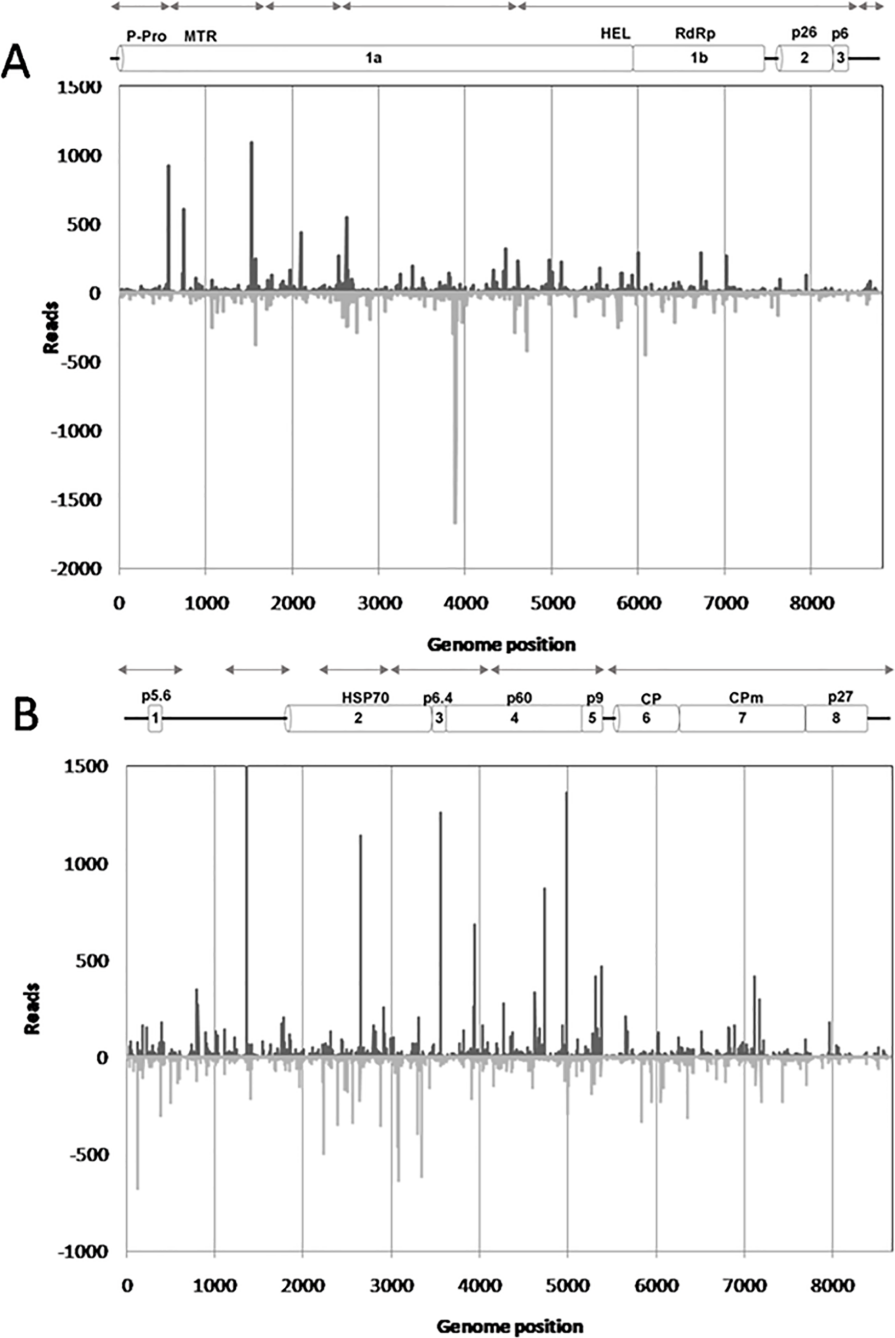
**Profiles distribution of 21-nt vsiRNAs along LCV-SP RNA1 (A) and RNA2 (B)**. Sense and antisense of 21-nt vsiRNAs orientations are above and below the axis respectively. Solid lines represent the location of contigs assembled from virus-derived small RNAs of LCV-SP. Schematic representation of LCV-SP RNA1 and RNA2, genome organization is represented as reference.

After removal of adapters and reads shorter than 18 nt, deep sequencing from small RNA library produced a set of 44,578 million reads, which were subjected to the novo assembly, generating 19,739 contigs. A total of 113 and 125 sequences of these contigs assembled with LCV-SP RNA1 and RNA2, respectively. Fig 2 shows the location of the contigs derived from small RNA reads and assembled to the LCV-SP RNA1 and RNA2 genome components. The recombinant genome of LCV-SP was confirmed also by deep sequencing library obtained from dsRNA extracted from a LCV-SP green bean-infected plant that resulted in 31,353,455 reads. These sequences were assembled resulting in 3642 contigs. These contigs covered 90% of the LCV-SP RNA1 genome and the consensus sequence result was 100% identical. Contigs assembled with LCV-SP RNA2 covered 94% of LCV-SP RNA2 genome with 99.7% coincidence.

The result of BLASTN is shown in S2 Table. Only sequences matching the RNA1 and RNA2 of different LCV isolates and the 3´end of the RNA1 of BnYDV (EU191904), corresponding to P26 and the 3´UTR, were identified. All the sequences identified in BLASTN as part of the genome of RNA1 of LCV (FJ FJ380118) showed high nucleotide identity (more than 80%) with ORFs 1a and 1b. No sequences corresponding to the 3´end of RNA1 of LCV were identified, confirming the presence of the first *Crinivirus* arising by crossover recombination of P26 and P6 belonging to the 3´end of RNA1 of BnYDV (EU191904) and discarding the presence of any Californian or Chinese-related LCV in the plants.

The profiles of vsiRNAs aligned to LCV-SP RNA1 and 2 were analysed. The 21-nt vsiRNA class population was most prevalent, followed by the 22-nt (10.1 and 2.6 million, respectively). From this 21-nt population, 58 675 and 80 904 reads matched to LCV-SP RNA1 and 2 genome. In the case of 22-nt class population, 53 932 and 68 146 matched with LCV-SP RNA1 and 2. The analysis of 21 and 22-nt vsiRNA strand polarity showed similar pictures, 53% of 21 and 22-nt vsiRNA class populations aligned to LCV-SP RNA1 were negative, whereas more than 50% of 21-nt and 22-nt vsiRNA class populations aligning to LCV-SP RNA2 were positive (53% and 51%, respectively). The profile distribution of both vsiRNAs populations aligning to the LCV-SP RNA1 and RNA2 genome components were also very similar. The distribution of 21-nt vsiRNAs along LCV-SP RNA1 and 2 genome is shown in the Fig 2. Several vsiRNA-generating regions (named as hot spots) were identified in both genomic RNAs of LCV-SP. Hot spots in RNA1 were located between MTR and HEL domains. The distribution of vsiRNA hotspots in the RNA2 genome corresponding with ORF 2 (HSP70), 3 (P6.4) and 4 (P60) are correlated with the position of subgenomic RNAs in LCV described by [6]. A hotspot located in the position 1361 of RNA2 does not correspond with any putative coding region.

### Phylogenetic analysis

**Fig 3:**
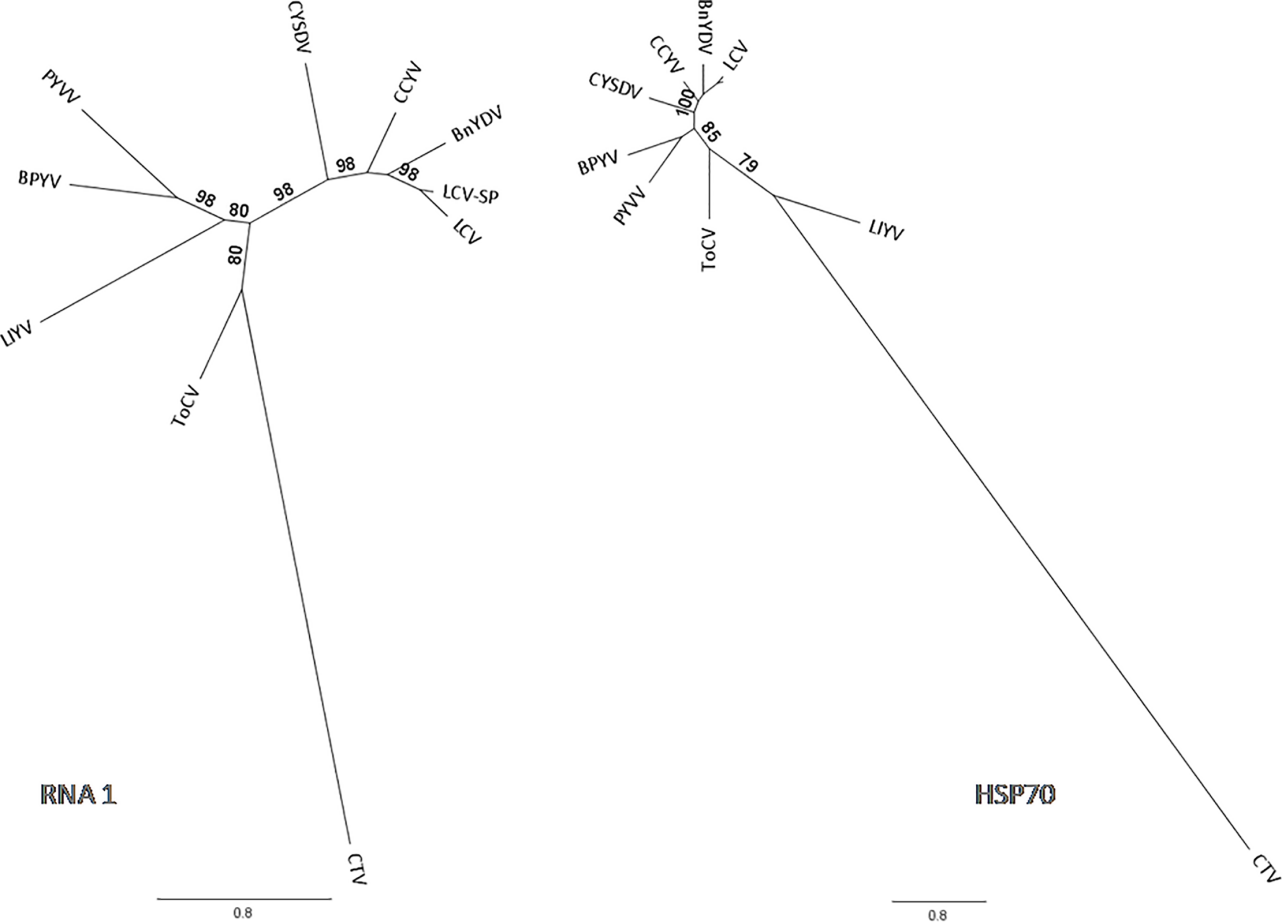
Bayesian phylogenetic trees of complete sequence of RNA1 and HSP70 region of RNA2 of selected viruses from the genus *Crinivirus*. *Citrus tristeza virus* (CTV, NC001661), was used as outgroup. *Lettuce chlorosis virus* (LCV, FJ380118, FJ380119), *Lettuce chlorosis virus*-SP (LCV-SP, MG489894, MG489895), *Lettuce infectious yellows virus* (LIYV, NC003617, NC003618), *Cucurbit yellow disorder virus* (CYSDV, NC004809, NC004810), *Cucurbit chlorotic yellow virus* (CCYV, JQ904628, JQ904629), *Bean yellow disorder virus* (BnYDV, EU191904; EU191905), *Beet pseudo yellows virus* (BPYV, AY330918, AY330919), *Tomato chlorosis virus* (ToCV, KP137100, KP137101), *Potato yellow vein virus* (PYVV, NC006062, NC006063). Posterior probability is indicated.

The phylogenetic trees generated with the complete sequence of RNA1 and HSP70 region of RNA2 of LCV-SP and other members of genus *Crinivirus* show two well differentiated lineages; one comprising LCV-SP, LCV, CCYV, BnYDV and CYSDV and the other is formed by BPYV, ToCV, PVYV and LIYV. Then, two clades were generated where the divergence is clear (Fig 3). Both phylogenetic trees display that, independently of the region analysed, BnYDV and CCYV are closely related to LCV-SP and LCV although LCV, LCV-SP and CCYV, have different host ranges and also have not been described in the same geographic area.

### Recombination analysis and variability in the recombinant genomic region

**Fig 4:**
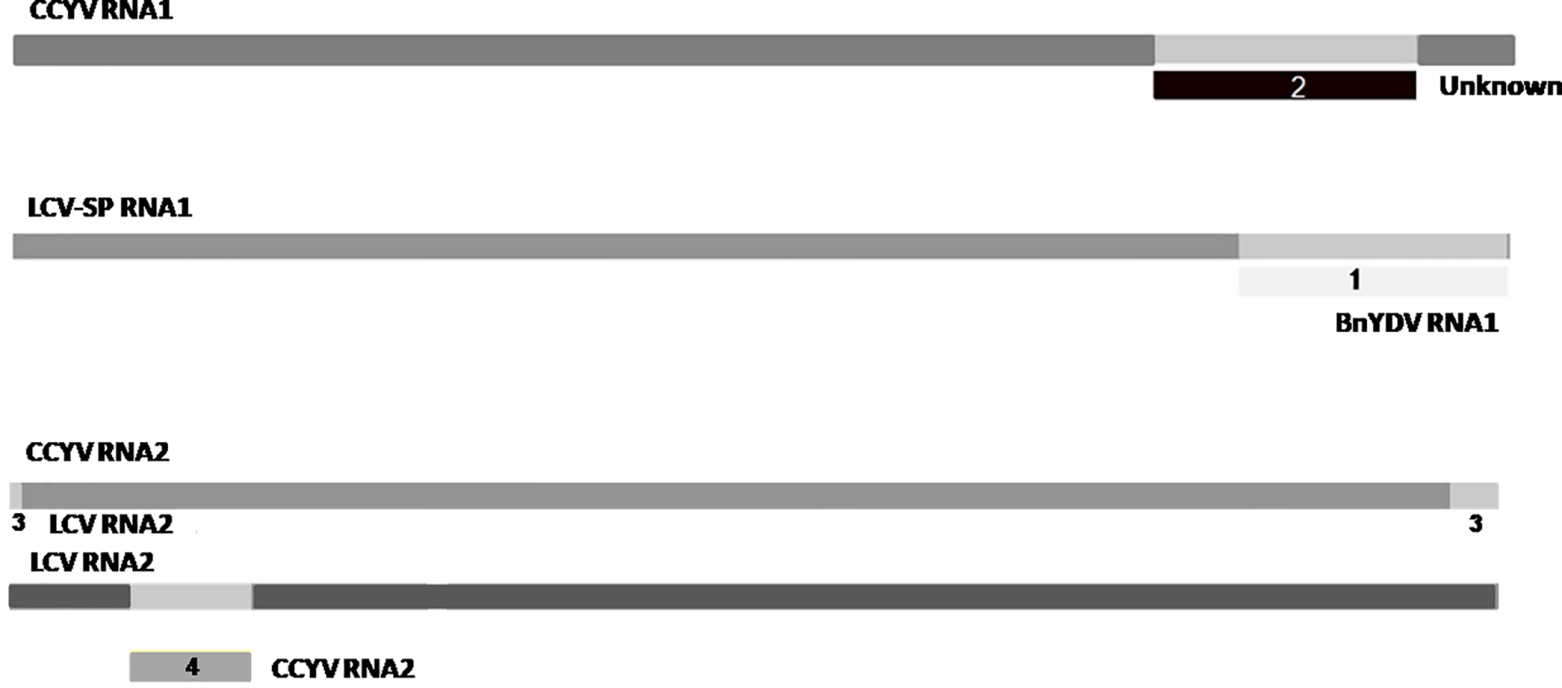
Recombination analysis of RNA1 and RNA2 of LCV, BnYDV, LCV-SP and CCYV using the recombination detection program (RDP-4). Four recombination events were detected in full-length genome RNA1 of LCV-SP and CCYV (events 1 and 2) and RNA2 of CCYV and LCV (events 3 and 4). Each RNA genome is indicated by a different color bar.

RDP4 package program was used to further confirm the putative existence of first recombinant of the genus *Crinivirus* detected by Sanger and deep sequencing. Aligned genomic sequence of RNA1 and RNA 2 belonging to LCV, BnYDV, CCYV and LCV-SP were scanned in RDP4 using multiple methods. The full recombination scan of both RNA genomes (1 and 2) of LCV, BnYDV, CCYV and LCV-SP resulted in four recombination events detected (Fig 4, Table 3). The analysis detected LCV RNA1 and BnYDV RNA1 as possible major and minor parental sequences for LCV-SP RNA1 (event 1) with a 99% level of confidence (Table 3, event 1). This event was identified by all seven methods implemented in this package and, all of them show lowest *p* value than any other algorithm executed in the rest of the events detected. BnYDV RNA1 was also suggested as a major parental sequence for CCYV RNA1 using five algorithms and with unknown minor parental (Table 3, event 2). Event 3 was detected by seven algorithms and signalized to LCV RNA2 as minor parental and CCYV-RNA2 as a possible recombinant. Although this event was suggested for seven methods as event number 1 was, the lowest *p*-value (7,181 x 10−^9^, RDP) is much higher than in event 1 (5,710x10-^103^, RDP). LCV RNA2 was identified for six methods as a putative recombinant in event 4 with LCV-SP RNA2 and CCYV RNA2 as major and minor possible parents respectively (Table 3, event 4). Also, percentages of nucleotide sequence identity between the recombinants LCV-SP RNA1 (Event 1) and LCV RNA2 (Event 4) and the parentals were analysed and showed high percentage of nucleotide identity (89.3 and 99.9) between the recombinant LCV-SP RNA1 and the parentals (S3 Table).

**Table 3:** Unique recombination events identified by recombination detection program v 4.80 (RDP4).

Event	Recombinant	Major parent	Minor parent	RDP	Geneconv	Bootscan	Maxchi	Chimaera	Siscan	3Seq	Begin	**End**
**1**	LCV-SP RNA1	LCV RNA1	BnYDV RNA1	5,710 x 10−^103^	6,418 x 10−^63^	2,665 x 10−^107^	5,521 x 10−^29^	4,462 x10-^28^	2,232 x 10−^36^	1,332x10-^15^	7580	8815
**2**	CCYV RNA1	BnYDV RNA1	Unknown	2,182 x 10−^4^			1,403 x 10−^2^	6,949 x 10−^4^	3,625 x 10−^5^	2,131 x 10−^2^	7044	8225
**3**	CCYV RNA2	Unknown	LCV RNA2	1,498 x 10−^9^	1,498 x 10−^4^	3,316 x 10−^9^	2,801 x 10−^2^	4,708 x 10−^4^	6,834 x 10−^6^	7,181 x 10−^9^	7858	75
**4**	LCV RNA2	LCV-SP RNA2	CCYV RNA2	6,581 x 10−^6^	2,614 x 10−^2^	6,247 x10-^3^	5,859 x 10−^9^	5,244 x 10−^6^		1,109 x 10−^4^	585	1046

To confirm the result obtained from RDP4, BootScan analysis using each putative recombinant as query sequence were executed with the Simplot package. When the analysis was performed for LCV-SP RNA1, a point of recombination was detected in the same genomic area described by RDP4 (S2 Fig). This result supports the evidence that LCV RNA1 (FJ380118) and BnYDV RNA1 (EU191904) are the origin of the LCV-SP RNA1 genome. The recombination events 2 and 4 however, were not recognized by the Simplot package. Bootscan analysis using CCYV RNA2 as query (S3 Fig) suggested a recombination point at 3´UTR as is described in RDP4 (Fig 4) but not in the 5´UTR region (S3 Fig).

DNA fragments obtained in surveys between 2011–2017 and corresponding with the entire P26 and P6 sequence of the RNA1 of LCV-SP were submitted to the GenBank database with the accession numbers assigned (MH170031-MH170041). These sequences confirmed the presence of the recombinant LCV-SP in the south of Spain since at least 2011. Values of genetic distance sequences at non-synonymous positions for both ORFs were of the same magnitude (Table 4). The ratio between nucleotide diversity values in non-synonymous and synonymous positions (dNS/dS ratio) indicates the amount of variation in the nucleic acid which results in variation in the encoded protein [41–43]. For P26, genetic distance at synonymous positions was null, and consequently, it was not possible to calculate the dNS/dS ratio. In the case of P6, The dNS/dS ratio was below unity, which could indicate a negative selection against protein change. However, the value of the standard error is too high to consider the data conclusively.

**Table 4:** Nucleotide diversity for the 3´end LCV-SP genome.

Sequence	dNS	SE	dS	SE	*D*	SE
**p26**	0.00126	0.00087	0	0	0.0009	0.00072
**p6**	0.00167	0.00166	0.00305	0.00321	0.00212	0.00153

Nucleotide diversities computed for nonsynonymous (dNS), synonymous (dS) and base (*D*) positions using the PBL method (see Methods). Standard errors (SE) were calculated by bootstrap method with 500 replicates.

### Molecular differentiation between yellowing induced by BnYDV or LCV-SP

**Fig 5:**
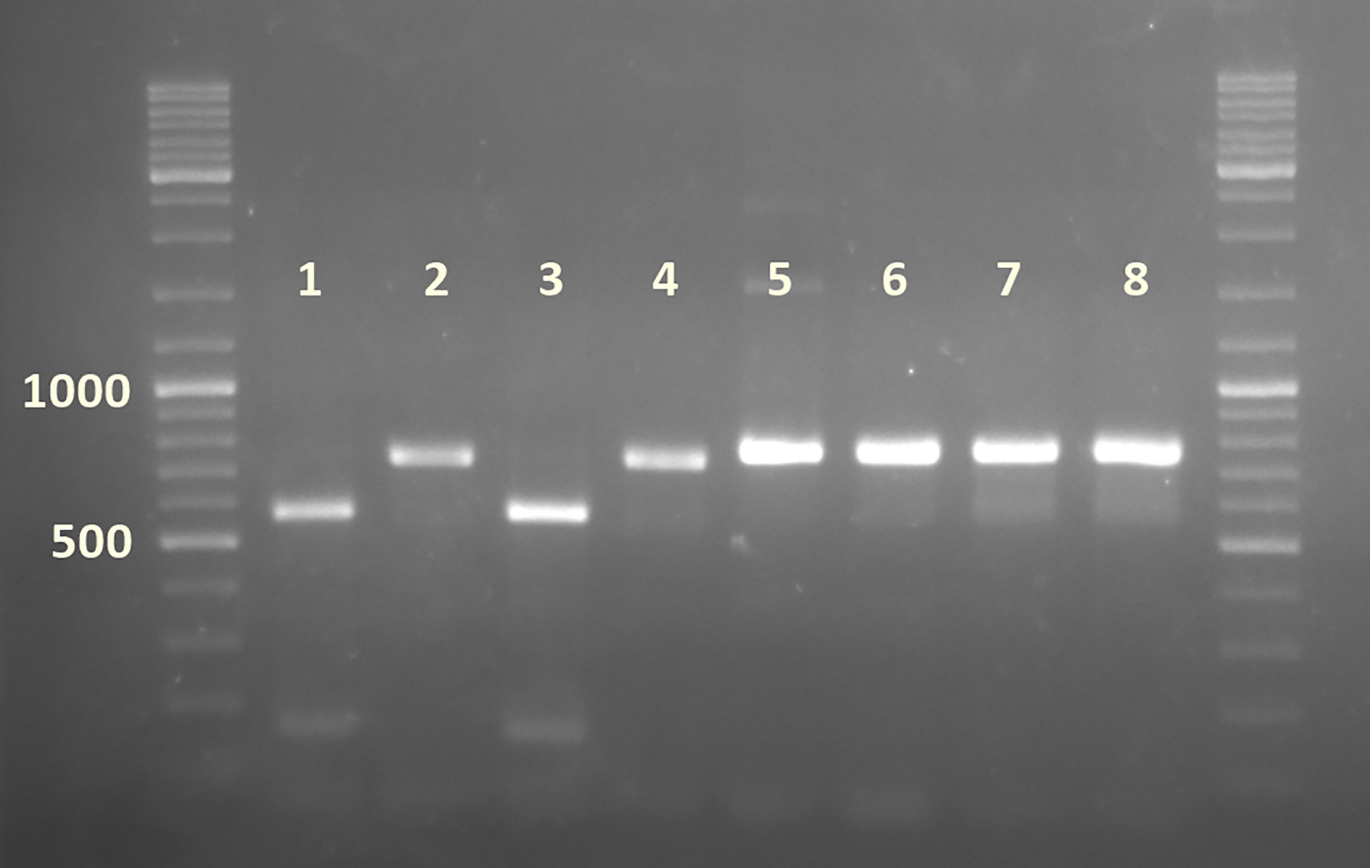
RFLP analysis with *Kpn*I after RT-PCR with by primers LC-BnF/LC-BnL. Lanes 1, 3: KpnI-digested amplification products of LCV-SP isolates obtained in 2013 and 2017 respectively. Lanes 2 and 4: same amplification products uncut. Lane 5 and 7: cut amplification product of BnYDV isolates obtained in 2005 and 2009. Lanes 6 and 8: same amplification products uncut.

Restriction analysis with *Kpn*I after RT-PCR with the primers LC-BnF/LC-BnL, which amplified partial sequence of RdRp and P26 in both viruses, yielded two fragments of 582 y 157 bp when the virus was LCV-SP and a single band of 769 bp when the virus present was BnYDV. This RFLP test is then capable of distinguishing yellowing disease originating from recombinant or non-recombinant virus isolates (Fig 5).

## Discussion

Members of genus *Crinivirus* represent worldwide emerging diseases as evidence of the increasing number of new species identified during the past 20 years [44–46, 9, 4]. Other members of the genus emerge, not as a new species, but as a new strain affecting a new host range [47]. New LCV isolates from China have been recently described affecting ornamental plants, tobacco and tomato crops [12,13,48]; but there is no information about the capability of these LCV isolates to infect lettuce crops. Primer walking method and deep sequencing of vsiRNA allowed the elucidation of the sequence and genome organization of the first recombinant closterovirus resulting after crossover recombination of intact ORFs. The 5´ from LCV-SP RNA1 includes the replication module (ORF 1a and 1b), which share high homology with the LCV type isolate (FJ380118); however, the 3´end, which encompass P26 and P6, shares high amino acid identity with the 3´end of BnYDV RNA1. These ORFs, are described to be expressed in LCV from 3´ coterminal subgenomic RNAs (sgRNAs), a typical strategy among closteroviruses [8,6,49] that increases the probability of recombination events [50]. The existence of this recombinant region in the genome of LCV-SP was confirmed by genome sequencing after primer walking, deep sequencing of vsiRNAs and dsRNA, and RT-PCR of genomic RNA from purified virion.

The genomic sequence of LCV-SP RNA2 shares 8 ORFs with the Californian isolate of LCV (FJ380119); P6, and P4.8, located in the 5´and 3´end of the Californian isolate, respectively, and with unknown function, are absent in LCV-SP. Both putative proteins are also absent in the LCV Chinese isolates recently reported [12,13], which could indicate that these genes are not essential in the biology of LCV.

3´UTRs sequences in both genomic RNAs are highly conserved, sharing 72% of nucleotide sequence identity. This feature is common among criniviruses [51] and it agrees with the notion that high sequence identity indicates that these sequences are essential for synthesis of the negative sense RNA during replication [52,53,49].

Higher number of 21-nt class vsiRNAs populations with respect to 22-nt support the prevalence of a Dicer like protein 4 (DCL4) in the production of vsiRNAs in beans, as has been suggested in others plants [54,55]. 21- and 22-nt vsiRNAs populations, showed the same preference for sense or antisense polarity in RNA1 and RNA2, indicating that the mechanisms responsible for strand polarity are not dependent on the preference of DCL enzymes but on other factors specific to this virus species. The nucleotides present at the 5’ and 3’ terminal positions were investigated and resulted in an overall preference for A/U nucleotides. This prevalence is widespread among plant virus-pathosystems [56] and indicates the involvement of similar AGO complexes in the silencing of LCV-SP by green beans. Although the function of P26 and P6 in LCV-SP RNA1 (and hence BnYDV-RNA1) is unknown, the reduction of vsiRNA populations in this region could suggest that P26 or P6 interfered with the accumulation of vsiRNA. Recently, P23 of LCV, the closest homologous protein of P26, and also located at the 3´end of RNA1, has been described as a viral suppressor of RNA silencing (VSRs) and whose suppressor activity produces a reduction of siRNA [7]. To elucidate this hypothesis additional experiments are required.

Recombination analysis completed with RDP4 and Simplot programs, demonstrates the existence of natural crossover recombination of intact ORFs between LCV-RNA1 and BnYDV-RNA1 (event 1). The recombinant virus, LCV-SP, shifted its host range by infecting green bean crops and not lettuce, its original host [11,5]. No other evident recombination events were detected in our analyses. Events 2, 3 and 4 have been discarded as possible recombinants although, alternatively they could be indicative of the close genetic relationship between LCV, LCV-SP, BnYDV and CCYV, as has been observed in the phylogenetic analysis. Recombination is important in positive-sense single-stranded RNA virus [18]. Plant viruses with segmented genomes seem to frequently accumulate recombinants, pseudorecombinants and reassortments [50, 57]. Recombination is considered a driving force of the emergence of plant viruses, showing changes of host range or virulence after recombination. Such adaptive recombination has been shown for single-stranded RNA and DNA viruses allowing for host shifts and increased host ranges, sometimes leading to new epidemics [58–60]. Perhaps, similar events explain why the recombinant virus, LCV-SP, has shifted its host range by affecting green bean crops and not lettuce, its original host [11,5]. Within genus *Crinivirus*, which shows the lowest variability within the family *Closteroviridae* [24], only *Sweet potato chlorotic stunt virus* (SPCV) and *Beet pseudo yellows virus* (BPYV) have been reported as examples of recombination-mediated gene gain [47,26]. The existence of LCV-SP changes the perspectives of the possible epidemiological scenarios within the genus *Crinivirus*, an emergent genus and, as we have demonstrated, with more recombination possibilities than previously shown.

## Supporting information

S1 Table Primers list used to confirm the LCV-SP genome by RT-PCR. (DOC)

S2 Table Numbers of reads aligning with a reference accession by BLASTN from contigs obtained from small RNAs and dsRNAs libraries. (DOC)

S3 Table Percentages of nucleotide sequence identity between the recombinants LCV-SP RNA1 (Event 1) and LCV RNA2 (Event 4) and the parentals. LCV-RNA1: FJ380118; BnYDV-RNA1: EU191904; LCV-SP-RNA2: MG489895; CCYV RNA2 904629. Parentals in the putative recombinants 2 and 3 are unknown. NA: not available.
(DOC)

S1 Fig RT-PCR identification of LCV-SP from virion purification. Lane 1: LCVSP-1F/LCVSP-2R; Lane 2: LCVSP-3F/LCVSP-4; Lane 3 LCVSP-54F/LC-Bn51R.
(TIF)

S2 Fig Bootscan analysis with Simplot program using LCV-SP RNA1 as the query sequence. (TIF)

S3 Fig Bootscan analysis with Simplot program using CCYV RNA2 as the query sequence. (TIF)
