# Supplementary material for: First natural crossover recombination between two distinct species of the family Closteroviridae leads to the emergence of a new disease
Source: PLoS One. 2018 Sep 13;13(9):e0198228. doi: 10.1371/journal.pone.0198228 (PMC6136708; doi:10.1371/journal.pone.0198228)
Supplement: S1 Table — (DOC) [file pone.0198228.s001.doc]

| **Name** | **Sequence 5´to 3´** | **Position** | **Sense** | **Segment** |
| --- | --- | --- | --- | --- |
| **LSP-346F** | AAGGCTAGTATCCCCTGGCA | 327-346 | Foward | RNA 1 |
| **LSP-1595R** | TCACTTCGAGTCTGTGTGCC | 1575-1595 | Reverse | RNA 1 |
| **LSP-1083F** | AGAACACCACCCCTCAGAGT | 1064-1083 | Foward | RNA 1 |
| **LSP-2214R** | AGTGAAGTCTACTCTCTTAGCTTTGT | 2099-2124 | Reverse | RNA 1 |
| **LSP-1943F** | GATGCACATCGGCACATGAC | 1924-1943 | Foward | RNA 1 |
| **LSP-2902R** | TACACTGGTTGGAAGCGACC | 2883-2902 | Reverse | RNA 1 |
| **LSP-2876F** | TGAGTGAGGTCGCTTCCAAC | 2876-2895 | Foward | RNA 1 |
| **LSP-4028R** | ACCCTGCTGCCTATCAACAC | 4009-4028 | Reverse | RNA 1 |
| **LSP-4006F** | TGGAATTGTGACAGCTCCCA | 3990-4006 | Foward | RNA 1 |
| **LSP-4797R** | CACAGCGTTATTCACCCTGC | 4778-4797 | Reverse | RNA 1 |
| **LSP-4467F** | GCGTGGGTAGAACCGAGAAA | 4448-4467 | Foward | RNA 1 |
| **LSP-5492R** | CAAACTGTGTGAGCAACCCG | 5473-5492 | Reverse | RNA 1 |
| **LSP-5487F** | TAAACCGGGTTGCTCACACA | 5468-5487 | Foward | RNA 1 |
| **LSP-6318R** | GGTCTAGCCCTCTCACCCTT | 6299-6318 | Reverse | RNA 1 |
| **LSP-6088F** | GCCCCAGGTCAAAATCTTGC | 6069-6088 | Foward | RNA 1 |
| **LSP-6987R** | ACCGGACACACCACAATCTC | 7006-6987 | Reverse | RNA 1 |
| **LSP-6645F** | CGACTTATGATCCTCCGGCC | 6664-6645 | Foward | RNA 1 |
| **LSP-7793R** | TTCTGAGAGCTGCTCTGCTG | 7794-7793 | Reverse | RNA 1 |
| **LSP-7624F** | GATTATATACGCGTGAGTTGTG | 7603-7624 | Foward | RNA 1 |
| **LSP-8759R** | ATATTAATGTAATTCTACGGTC | 8738-8759 | Reverse | RNA 1 |
| **LSP2-192F** | AGCACACAGCTGTCAACCTG | 173-192 | Foward | RNA 2 |
| **LSP2-1348R** | CTCAGGCACTACATCGACGC | 1365-1384 | Reverse | RNA 2 |
| **LSP2-984F** | CGCCGTTCGAATATAACGTCG | 964-984 | Foward | RNA 2 |
| **LSP2-2430R** | CAGAGACGAGTCATACGTACC | 2410-2430 | Reverse | RNA 2 |
| **LSP2-2323F** | GGGTTTTTCGCTCAGGAGGA | 2304-2323 | Foward | RNA 2 |
| **LSP2-3569R** | ACTTGACCCTCAAACAAACGA | 3549-3569 | Reverse | RNA 2 |
| **LSP2-3320F** | GTCGAATGTGGATGAGACTGC | 3300-3320 | Foward | RNA 2 |
| **LSP2-4368R** | TCTGGACTCTGTACCATGGATTC | 4356-4378- | Reverse | RNA 2 |
| **LSP2-4223F** | CTACTGGCGCGTCAGTTGTA | 4204-4223 | Foward | RNA 2 |
| **LSP2-5267R** | CACCAGCAGACCATTTTGCC | 5248-5267 | Reverse | RNA 2 |
| **LSP2-5166F** | CACAAAACGAGACGAGTGCG | 5147-5166 | Foward | RNA 2 |
| **LSP2-6266R** | TCCTTTCAAAGCCTGGCACT | 6207-6226 | Reverse | RNA 2 |
| **LSP2-6082F** | AAGCCACGGGTAAGGTCAA | 6063-6082 | Foward | RNA 2 |
| **LSP2-7105R** | TCCGGAAATGGTGGAATCGG | 7124-7105 | Reverse | RNA 2 |
| **LSP2-7085F** | GACAATTTGAAACCGGGCGG | 7066-7085 | Foward | RNA 2 |
| **LSP2-8101R** | CCCGAATCTGGCATCGGTAA | 8082-8101 | Reverse | RNA 2 |
