# Supplementary material for: First natural crossover recombination between two distinct species of the family Closteroviridae leads to the emergence of a new disease
Source: PLoS One. 2018 Sep 13;13(9):e0198228. doi: 10.1371/journal.pone.0198228 (PMC6136708; doi:10.1371/journal.pone.0198228)
Supplement: S2 Table — (DOC) [file pone.0198228.s002.doc]

| **GenkBank** | **Acronym** | **sRNAs** | **dsRNAs** |
| --- | --- | --- | --- |
| FJ380118 | LCV | 7 | 11 |
| FJ380119 | LCV | 5 | 1 |
| KC602375 | LCV-SP | 5 | 13 |
| KC602376 | LCV-SP | 7 | 5 |
| KX685958 | LCV-NJ | 8 | 7 |
| KX685959 | LCV-NJ | 11 | 14 |
| KY271955 | LCV-PTX | 51 | 67 |
| KY271956 | LCV-PTX | 49 | 46 |
| KY430285 | LCV-CN | 15 | 20 |
| KY430286 | LCV-CN | 23 | 32 |
| EU191904 | BnYDV | 10 | 12 |
| Total Reads |  | 181 | 228 |
