# Supplementary material for: First natural crossover recombination between two distinct species of the family Closteroviridae leads to the emergence of a new disease
Source: PLoS One. 2018 Sep 13;13(9):e0198228. doi: 10.1371/journal.pone.0198228 (PMC6136708; doi:10.1371/journal.pone.0198228)
Supplement: S3 Table — LCV-RNA1: FJ380118; BnYDV-RNA1: EU191904; LCV-SP-RNA2: MG489895; CCYV RNA2 904629. Parentals in the putative recombinants 2 and 3 are unknown. NA: not available. (DOC) [file pone.0198228.s003.doc]

|  | Recombinant | |
| --- | --- | --- |
| Parentals | LCV-SP RNA1 | LCV RNA2 |
| LCV RNA1 | 89.3 | NA |
| BnYDV RNA1 | 99.9 | NA |
| LCV-SP RNA 2 | NA | 88 |
| CCYV RNA 2 | NA | 50 |
