# Supplementary figures and images for: First natural crossover recombination between two distinct species of the family Closteroviridae leads to the emergence of a new disease
Source: PLoS One. 2018 Sep 13;13(9):e0198228. doi: 10.1371/journal.pone.0198228 (PMC6136708; doi:10.1371/journal.pone.0198228)

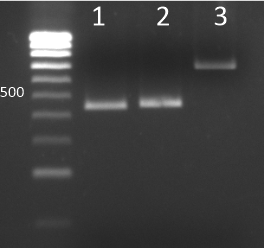

Supplement: S1 Fig — Lane 1: LCVSP-1F/LCVSP-2R; Lane 2: LCVSP-3F/LCVSP-4; Lane 3 LCVSP-54F/LC-Bn51R. (TIF) [file pone.0198228.s004.tif]

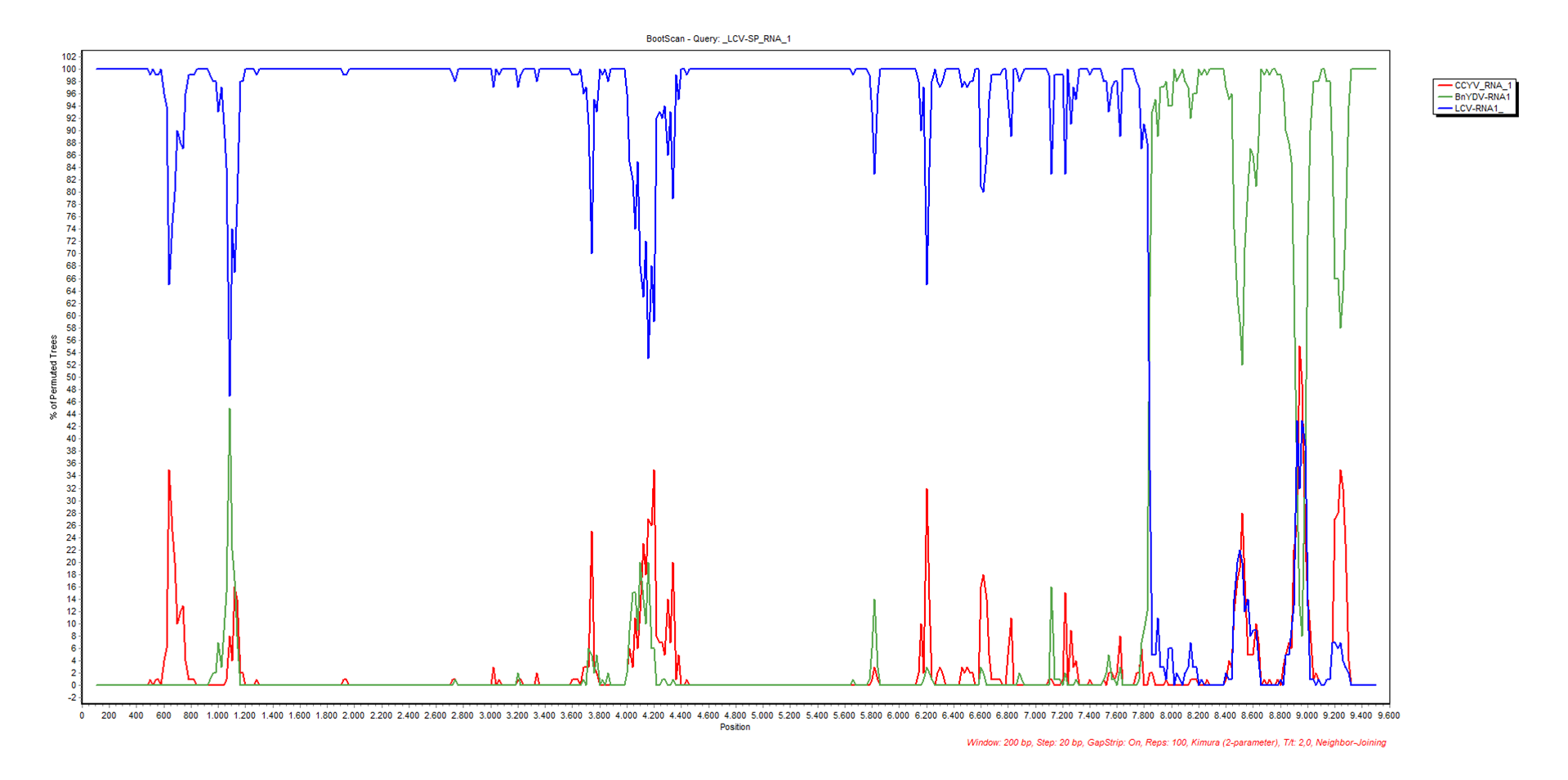

Supplement: S2 Fig — (TIF) [file pone.0198228.s005.tif]

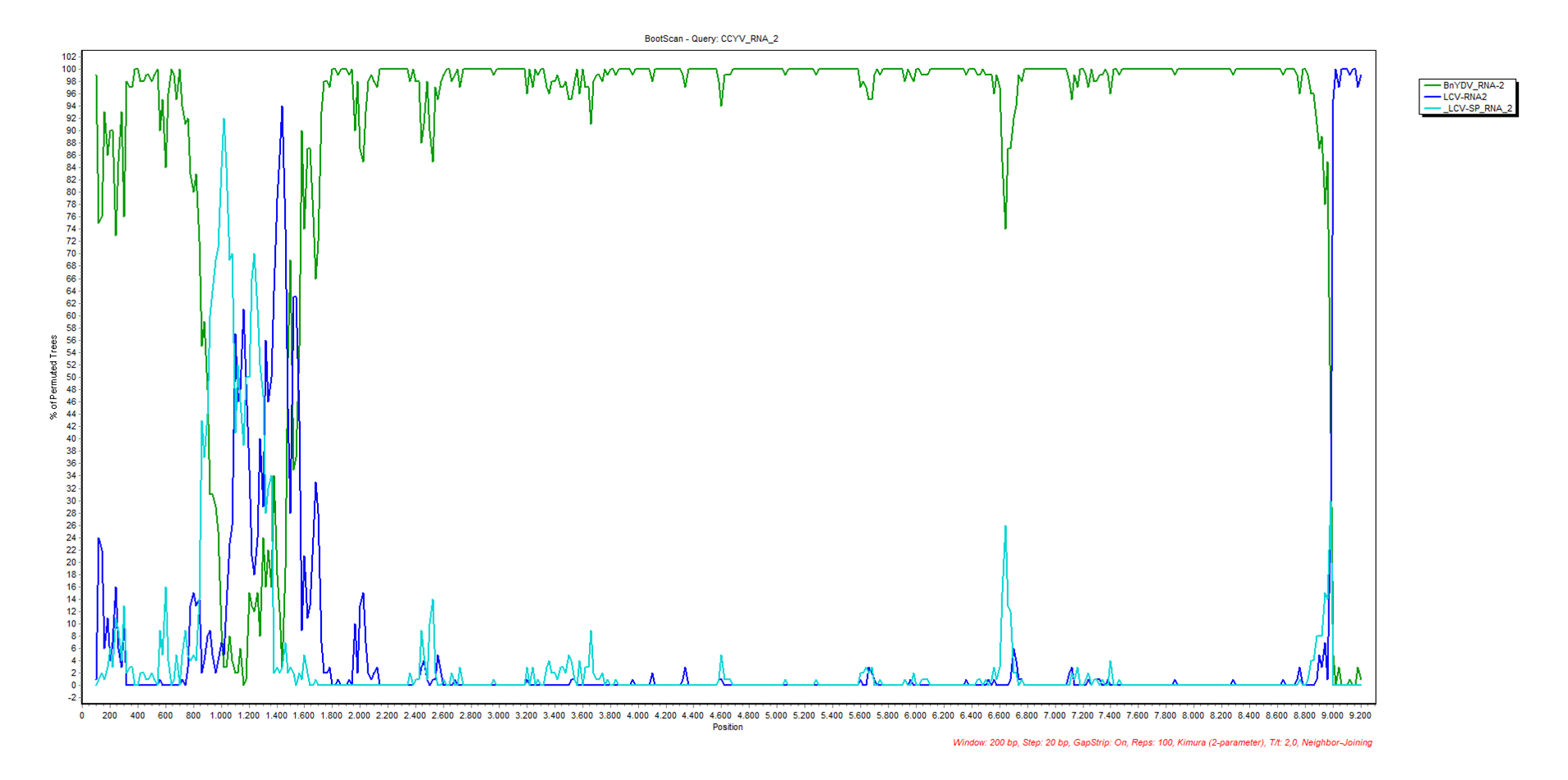

Supplement: S3 Fig — (TIF) [file pone.0198228.s006.tif]
